# Topical diacerein for epidermolysis bullosa: a randomized controlled pilot study

**DOI:** 10.1186/1750-1172-8-69

**Published:** 2013-05-07

**Authors:** Verena Wally, Sophie Kitzmueller, Florian Lagler, Angelika Moder, Wolfgang Hitzl, Martin Wolkersdorfer, Peter Hofbauer, Thomas K Felder, Michael Dornauer, Anja Diem, Nora Eiler, Johann W Bauer

**Affiliations:** 1 Division of Experimental Dermatology and EB House Austria, Department of Dermatology, Paracelsus Medical University Salzburg, Muellner Hauptstraße 48, Salzburg 5020, Austria; 2 Institute for Inborn Errors of Metabolism and Department of Pediatrics, Paracelsus Medical University, Salzburg, Austria; 3 Paracelsus Medical University, Research Office, Biostatistics, Salzburg, Austria; 4 Department of Production, Hospital Pharmacy, Landesapotheke, Salzburg, Austria; 5 Department of Laboratory Medicine, Paracelsus Medical University, Salzburg, Austria

**Keywords:** Epidermolysis bullosa, Small molecule therapies, Pilot study, Keratin 14, Diacerein, Interleukin-1ß

## Abstract

Blistering in epidermolysis bullosa simplex type Dowling-Meara (EBS-DM) is associated with an inflammatory phenotype, which can be disrupted by diacerein *in vitro*. In this pilot study we hypothesized, that a topical formulation of diacerein 1% reduces blistering. Five patients initially applied diacerein underneath both armpits. Then, each participant received 1% diacerein-cream for one armpit, and placebo for the other (randomized withdrawal). The number of blisters was reduced significantly (left: -78%; right: -66% of baseline) within two weeks and remained significantly below the initial level even during withdrawal in four patients. These findings point to a relevant effect of diacerein and provide important information for a confirmative study.

## Findings

EBS-DM is the consequence of dominantly inherited mutations in either the keratin 5 (*K5*) or keratin 14 (*K14*) gene, which encode proteins constituting the intermediate filament (IF) network of basal keratinocytes. Mutations lead to an increased mechanical susceptibility of keratinocytes, manifesting in a collapse of the IF network [1]. Clinically, patients suffer from blistering of the skin upon minor trauma, resulting in an impaired quality of life due to pain and pruritus [2]. *In vitro* studies on EBS-DM keratinocytes showed a significant upregulation of interleukin-1beta (IL-1ß), resulting in the activation of the c-jun N-terminal-kinase (JNK) stress pathway and subsequently in the overexpression of K14 and IL-1ß in a positive feedback loop. When impairing IL-1ß signaling, using anti-IL-1ß antibody or the small molecule diacerein, levels of IL-1ß, JNK and K14 decreased and the IF network was stabilized [3]. Based on this information, we launched a double-blinded, randomized, placebo-controlled pilot study using a 1% diacerein in the commonly used care cream ultraphil® as intervening agent, and ultraphil® alone as placebo.

Diacerein, a prodrug of the IL-1 converting enzyme inhibitor rhein, is approved for the systemic treatment of osteoarthritis (Verboril®) [4,5]. It is metabolized in the liver and cleared by the kidneys. Up to now there are no data available on topical application.

**Figure 1:**
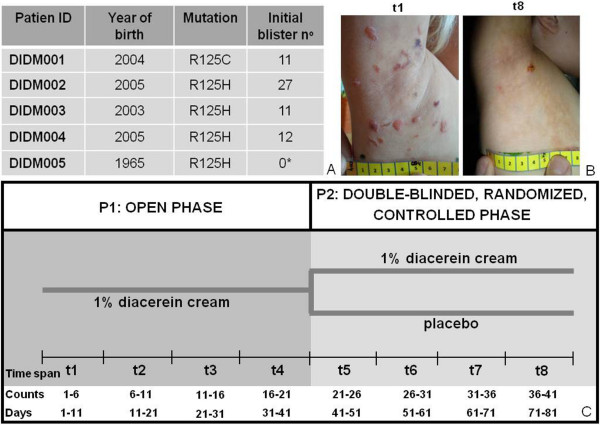
**Study design comprising an open phase and a controlled-randomized, double-blinded withdrawal.** (**A**) Five patients were included in the pilot study, all of them harboring a mutation in K14 protein position 125. Initial blister numbers are given. *Note that, contradictory to an aforegoing patient inquiry, patient DIDM005 did not have any blisters at the first measurement. (**B**) Representative pictures of the right armpit of DIDM002 during t1 and t8. (**C**) The pilot study was a two-phase study with a 6 weeks open phase (P1), followed by a six-weeks double-blinded, randomized and controlled phase (P2). During P1, patients daily applied 1% diacerein cream underneath both armpits. During P2, one armpit of each patient was assigned to placebo in a double-blinded and randomized manner. For further data analysis, the study was divided into eight equidistant time spans (t), each comprising six counts. Patients counted blisters every second day, starting from day 1.

**Figure 2:**
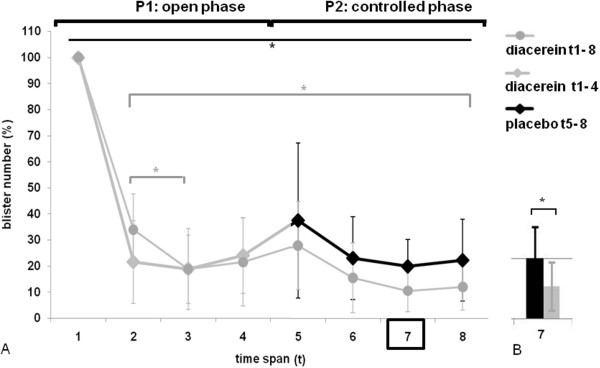
**Results of blister counts show a significant reduction during P1.** Patients received diacerein (grey graphs) to apply underneath both armpits during the open phase P1. During P2, one armpit was assigned to placebo (black graph) in a double-blinded, randomized manner. (**A**) During P1, there a significant reduction of blister numbers (given in%) from time span t1 to time span t2 and from t2 to t3 (t1:100%, t2:mean left: 22%/CI6-38; mean right: 34%/CI20-48). On both sides, t2 to t8 show a significant reduction of blisters compared to t1 (upper black line, * indicating a p-value ≤ 0.05). Blister numbers remained low until the end of the study period. The mean of blister numbers is given with 95% confidence intervals, whereby t1 is normalized to 100%. (**B**) During P2, there is a significant difference between placebo and diacerein at t7 (* indicates a p-value ≤ 0.05).

To test our hypothesis that diacerein is able to reduce blister formation, we recruited five patients (aged 6 to 48) with the diagnosis of EBS-DM and a heterozygous amino acid exchange in K14 protein position 125. An a-priori sample size computation was done in consideration of a 0.90 power, a 5% significance level and a minimum difference between blister numbers of 30%. A 25% standard deviation for the intervention group and 15% for the placebo group was expected. Computation was done for a one-sided, paired student t-test. Including a 10% drop-out rate, the calculation resulted in ten armpits of five patients to be included (Figure 1A). Randomization was done using random numbers, with the general requirement that no patient receives placebo or diacerein on both sides. The two-phased study started with a six-weeks open phase (P1), where all patients received 1% diacerein cream to apply underneath both armpits every evening. Usual care was continued unchanged. Blister numbers were documented every second day by the patients and every second week by a study nurse, who visited the patients at their homes to avoid any influences from travelling or changes of habits. Furthermore, patients were requested to take photos of the blisters plus a tape measure to allow for software aided quantification of blister areas (Figure 1B). To optimize the strength of our small pilot trial we added a second, randomized, placebo-controlled six-weeks phase (P2), exploiting the fact that placebo and verum could be used in parallel in the same patient, which virtually doubled our sample size. Time to loss of efficacy (defined as halving the effect in P1) was chosen as primary endpoint, because this allows minimizing the time on placebo, as patients can be de-blinded and switched to verum as soon as loss of efficacy occurs. For data analysis, we sub-divided the study period into eight time intervals (t), each including six counts (Figure 1C). Patient DIDM005 (1965) did not develop blisters during the study, contradictory to an aforegoing patient inquiry on times of most strain. DIDM005 was therefore excluded from the statistical evaluation. However, it cannot be excluded that the absence of blisters is due to the treatment. As blister numbers are likely to be correlated (two measurements within the same subject), generalized estimation equations (GEE) were used. The group (verum/placebo) was the main effect and time*group was used as interaction effect. The robust method (Huber/White/sandwich estimator) was used for estimating the covariance matrix. Pairwise comparisons are based on the sequential Bonferroni method. The significance level was set to 5%. Results showed a statistically significant reduction of blisters within the first two weeks of P1 (t1: 100%, t2: mean left: 22%/CI6-38; mean right: 34%/CI20-48), which remained stable until the end of the study period (Figure 2A). In P2, we could not observe any loss of efficacy and missed our primary endpoint. Although the relative number of blisters was slightly higher on the placebo sides, there was only a statistically significant difference at t7 (Figure 2B). We believe that carrying-over of the positive effect from P1 into P2 is the reason for that observation. Thus, unless missing the primary statistical objective, the results point to a beneficiary effect of diacerein.

Clearly, our study is only a pilot and limited by the low number of patients and the fact that the primary endpoint was missed. Still, it is a solid basis for planning an international multi-center study with the aim to confirm efficacy and safety. The conclusion that will be regarded in the design of this study comprises:

(a) The chosen dose and treatment regimen can lead to substantial reduction of blisters within 2 weeks.

(b) This effect seems to be rather stable and can persist for several weeks after cessation.

(c) Thus, it seems reasonable to include only patients with a substantial blistering at study entry and study the reduction of blisters in a randomized controlled manner.

(d) Furthermore, assessment of blistering should be done by study staff, because the validity of self-evaluation showed to be critical, although patients received a training in blister counting and foto-documentation.

## Consent

Written informed consent was obtained from the patients or the patient's parents for publication of this report and any accompanying images.

## Competing interests

The authors declare that they have no competing interests.

## Authors’ contributions

VW: Project coordination, conception and design, or acquisition of data, or analysis and interpretation of data. SK: data analysis. FL: conception and design, monitoring. AM: conception and design, monitoring. WH: Statistical analysis and interpretation of the data. MW, PH: Diacerein cream and placebo production, blinding, randomization. TKF: Diacerein analysis by LC-MS/MS. MD: Data aquisition and patient mentoring. AD, NE: Patient mentoring. JWB: Sponsor investigator. All authors read and approved the final manuscript.
